# The Study of the Optimal Parameter Settings in a Hospital Supply Chain System in Taiwan

**DOI:** 10.1155/2014/967140

**Published:** 2014-08-27

**Authors:** Hung-Chang Liao, Meng-Hao Chen, Ya-huei Wang

**Affiliations:** ^1^Department of Health Services Administration, Chung Shan Medical University, No. 110, Section 1, Jian-Koa N. Road, Taichung 402, Taiwan; ^2^Department of Medical Management, Chung Shan Medical University Hospital, No. 110, Section 1, Jian-Koa N. Road, Taichung 402, Taiwan; ^3^Department of Applied Foreign Languages, Chung Shan Medical University, No. 110, Section 1, Jian-Koa N. Road, Taichung 402, Taiwan; ^4^Department of Medical Education, Chung Shan Medical University Hospital, No. 110, Section 1, Jian-Koa N. Road, Taichung 402, Taiwan

## Abstract

This study proposed the optimal parameter settings for the hospital supply chain system (HSCS) when either the total system cost (TSC) or patient safety level (PSL) (or both simultaneously) was considered as the measure of the HSCS's performance. Four parameters were considered in the HSCS: safety stock, maximum inventory level, transportation capacity, and the reliability of the HSCS. A full-factor experimental design was used to simulate an HSCS for the purpose of collecting data. The response surface method (RSM) was used to construct the regression model, and a genetic algorithm (GA) was applied to obtain the optimal parameter settings for the HSCS. The results show that the best method of obtaining the optimal parameter settings for the HSCS is the simultaneous consideration of both the TSC and the PSL to measure performance. Also, the results of sensitivity analysis based on the optimal parameter settings were used to derive adjustable strategies for the decision-makers.

## 1. Introduction

An optimal supply chain (SC) is a strategy plan that covers the network of suppliers, hospital's manufacturing factors, and customers. An optimal SC will decrease the costs of material flow from suppliers to customers. Hence, most of the literature focuses on SC costs. García-Dastugue and Lambert [1] established an Internet-based system in order to integrate business processes using the flow of information to improve business practices in the SC. The big advantage of the Internet-based system was the opportunity it offered to purchase some items at a lower price. Wan et al. [2] used a simulation-based optimization framework for the manufacturing processes associated with SC. They used two small examples to simulate a three-stage SC for the function of total cost and to obtain the optimal inventory levels. Their results showed that the framework can accommodate chance constraints and scales up well. Yao et al. [3] explored the optimization of the ordering process in considering the minimization of a collaborative SC's total costs by using a vendor-managed inventory. van der Vlist et al. [4] revised Yao et al.'s model [3] by considering redistributing risk, coordinating replenishment for multiple buyers, sharing downstream demand data, a choice of stock positioning, and giving suppliers the latitude to change delivery times and the authority to adjust delivery quantities in response to inventory developments. Thus, the use of the SC model could optimize total costs when coordinated inventory management is implemented. Frota Neto et al. [5] developed a sustainable logistics network and discussed how to evaluate the effects of the trade-off between the costs of the logistics network and its environmental impact. Ganga and Carpinetti [6] proposed a SC model and used fuzzy logic to forecast its performance as to cost, responsiveness, reliability, and so forth. They showed that fuzzy logic is a feasible technique that is helpful in managing SC performance.

Most studies of SCs are in the industrial field; only a few have focused on the hospital supply chain system (HSCS). Schut and van Bergeijk [7] indicated that hospitals in most developing countries have high medical costs and suggested that they should go through a centralized government agency to assist purchasing in order to decrease the general price level of pharmaceuticals. Shah [8] researched a strategy to optimize SC in the pharmaceutical industry and concluded that the pharmaceutical industry needs to balance future customer demand with the production planning necessitated by clinical and competitor activity. Hence, effective capacity utilization planning and robust infrastructure investment are important key issues. Lapierre and Ruiz [9] studied an innovative approach for helping hospitals improve their scheduling logistics by coordinating procurement and distribution operations while respecting inventory capacities. Ghandforoush and Sen [10] presented a decision support system for platelet production SC with flexible scheduling. They suggested that, to meet daily demand, the decision support system should implement a superior production and mobile assignment schedule.

On the other hand, in order to plan a SC with mathematical precision, many studies used an experimental design to construct the model. Holweg et al. [11] used the Taguchi method and a simulation of a multitier SC to investigate the impact of scheduling activities. They concluded with a set of recommendations on how to improve current vehicle supply systems by forecasting scheduling systems. Delavar et al. [12] proposed a SC to coordinate production scheduling and air transportation. A Taguchi experimental design was applied to obtain the parameter values that would best improve their performance. Tiwari et al. [13] addressed the problem of an integrated SC design, and in order to ensure high service levels, a novel algorithm combined the Taguchi technique with an Artificial Immune System that was used to solve the problem. Yang et al. [14] improved the robustness of SC information-sharing strategies using a hybrid Taguchi method. Their results showed that when customer demand is uncertain, e-shopping has the most robust performance.

Based on the above literature review and significance, the purpose of this study is to design and plan a robust HSCS. This study used a simulated HSCS to analyze the different factors involved and to decide which parameters would affect the performance of the HSCS. These parameters were analyzed and used to create a robust design for the HSCS.

The simulated HSCS used in this study was based on computation technology, which allowed for the construction of a dynamic system that included information acquisition, processing, and management. The computation technology included a full-factor experimental design and response surface method (RSM), and a genetic algorithm (GA) was applied here to obtain the optimal parameter settings for the HSCS. Three responses, including total system cost (TSC), patient safety level (PSL), and overall performance (OP), were considered in the simulated HSCS. This study also obtained the optimal parameter settings for the robust HSCS and explored the sensitivity analysis of the HSCS's OP to derive the adjustable strategies. In addition, different weight combinations for exploring the trade-off effects of TSC and PSL were discussed.

The innovation of this paper is the use of the RSM and GA method to create the HSCS and to obtain the robust parameters' setting. Based on the literatures, the authors cannot find the research using RSM to design the HSCS. So this is the knowledge gap this work aims to cover.

## 2. The Problem of HSCS Parameter Settings

In this paper, three regional hospitals formed an HSCS alliance that included suppliers and a centralized purchasing center (CPC). Centralized purchasing allows large orders to be placed, which may result in a discounted price for each hospital, thus giving them a competitive advantage by reducing each participating hospital's total costs. However, the parameters used to coordinate the HSCS for centralized purchasing and distribution are very important. With robust parameter settings, the HSCS could attempt to minimize the TSC and maximize the PSL. In order to obtain robust HSCS parameter settings, the scenario for the HSCS is described below.

Three hospitals forecast the demand for clinical masks for each season based on past data. The demand random variable *D*_*lm*_ (uncertain demand in each period *m* for hospital *l*) is the normal probability distribution *N*(*μ*_*l*_, *σ*_*l*_^2^), in which *μ*_*l*_ is the mean (units/month) and *σ*_*l*_^2^ is the variance (units^2^/month^2^). Here, the *D*_*Am*_ for Hospital *A* is *N*(460,100^2^), *D*_*Bm*_ for Hospital *B* is *N*(310,90^2^), and *D*_*Cm*_ for Hospital *C* is *N*(400,83^2^). Because the *D*_*lm*_ for each hospital is independent of that of the others, the CPC collects the total demand from the three hospitals to calculate the random variable of the total ordering quantity and aggregates the three hospitals' normal probability distribution $N(\sum_{l=A}^{C}\mu_{l},{\left(\sqrt{\sum_{l=A}^{C}\sigma_{l}^{2}}\right)}^{2})$. After aggregating the forecast demands, the CPC submits the total order quantity to a pharmaceutical company. The pharmaceutical company then delivers quantities according to the hospitals' orders, meeting each hospital's needs. The evaluation indexes of the HSCS's performances are TSC and PSL. TSC is procurement cost + inventory cost + transportation cost. The formulation is as follows:

(1) TSC=∑H=AC∑m=112(Qm∗Pm∗DI+EIm∗Pm∗ICCRm+TQm∗TPm).

TSC is the total system cost of the HSCS. *H* is Hospital *A*, *B*, or *C*. *m* is the period from the 1st to the 12th month. *Q*_*m*_ is the distributed quantity for period *m*. *P*_*m*_ is the procurement cost for each unit in period *m*; it is set to normal probability distribution *N*(5,0.78^2^). DI is the discount rate for the total ordering quantity; it is set to normal probability distribution *N*(0.98,0.2^2^). EI_*m*_ is the inventory level for period *m*. ICCR_*m*_ is the rate of the holding cost per unit for period *m*; it is set to normal probability distribution *N*(0.31,0.09^2^). TQ_*m*_ is the number of times orders are transported from the pharmaceutical company to the hospitals. TP_*m*_ is the pharmaceutical company's shipping cost per time to the hospitals. When the transportation capacity is 500 units, it is set to normal probability distribution *N*(3400011500^2^). When the transportation capacity is 230 units, it is set to normal probability distribution *N*(16500,4500^2^). When the transportation capacity is 100 units, it is set to normal probability distribution *N*(7550,140^2^).

In addition, the HSCS's PSL is broken down as follows. Because there is a gap between forecasted demand and actual demand, the material flow of the HSCS [15–18] may result in a shortage and by extension affect patient safety. Hence, avoiding a shortage is another aspect of an HSCS's performance. Service level can thus be a feasible index to measure quantity shortages. In this paper, the service level is the index of PSL [19].

The PSL is defined as “1 − the shortage of quantity/the actual demand,” formulated as follows:

(2) $$\begin{array}{c} \text{PSL}=1-\frac{\sum_{H=A}^{C}\sum_{m=1}^{12}\text{S}{\text{O}}_{m}}{\sum_{H=A}^{C}\sum_{m=1}^{12}D_{m}}, \end{array}$$

where SO_*m*_ is the shortage quantity for period *m* and *D*_*m*_ is the actual demand for period *m*.

The inventory and shortage level are defined in (4):

(3) $$\begin{array}{c} \text{E}{\text{I}}_{m-1}+Q_{m}-D_{m}=\text{E}{\text{I}}_{m}-\text{S}{\text{O}}_{m}. \end{array}$$

The following equation shows the quantity *Q*_*m*_, which is subject to the safety stock (*Q*_min⁡_) and the maximal level of inventory (*Q*_max⁡_):

(4) Qm={0,Qmin⁡≤EIm−1≤Qmax⁡Qmax⁡−EIm−1,EIm−1>Qmin⁡,for  t=1,2,…,T.

From (1)~(4), we obtain the decision variable, *Q*_*m*_.

The HSCS's performances—TSC and PSL—will result in a trade-off effect because, by obtaining larger quantities, PSL will improve because of a decrease in the probability of an inventory shortage; however, the inventory will increase TSC.

The safety stock, maximum inventory level, transportation capacity, and HSCS reliability were selected as the parameters for the robust HSCS. Also, the four parameters affect the TSC and PSL. Generally, an increase/decrease of safety stock or maximum inventory level will cause an increase/decrease of TSC and PSL. An increase of transportation capacity will cause a decrease of transportation times; it will also affect the stock quantity and later affect the TSC and the PSL. The reliability of supply chains is defined as the ability to perform the promised service of customer expectations [20, 21]. Here, the performances include TSC and PSL, which are based on the distributed quantity. Hence, the reliability of HSCS is defined as the ratio of quantities supply in time for different suppliers.  Table 1 shows the different levels of these parameters.

To solve this HSCS problem, this paper used a full-factor experimental design in which 81 (3^4^ = 81) combinations of parameter levels were simulated. Each combination was simulated 1000 times in order to reduce the variation of the means of the TSC and the PSL; the means of the TSC and the PSL were then calculated for each combination. In addition, because the TSC and the PSL are simultaneously considered here, to help the decision-makers to set the robust parameters' level, it is necessary to transfer the two responses to one multiresponse objective. Hence, in order to integrate TSC and PSL into one OP, the ideal function was adopted here.

The normalized mean of TSC for each combination (TSC_mean_) is defined as NTSC = TSC_min⁡_/TSC_mean_, where TSC_min⁡_ is the minimization of TSC_mean_. Thus, the larger the NTSC the better, and the value is between 0 and 1.

The normalized mean of PSL for each combination (PSL_mean_) is defined as NPSL = PSL_mean_/PSL_max⁡_, where PSL_max⁡_ is the maximization of PSL_mean_. Thus, a larger NPSL is again better, and the value is between 0 and 1.

The OP is defined by the ideal function; hence, $\text{OP}=\sqrt{\text{NTSC}*\text{NPSL}}$. Thus, the larger the OP the better, and the value is between 0 and 1.

The response surface method (RSM) [22] was applied here in order to obtain the regression model using the parameters and the responses—NTSC, NPSL, and OP. Because the RSM is a model building technique using statistical experimental design and least square error fitting, it can be used to approximate a response function in terms of predictor variables. The approximation of the response functions for the available data is set as follows. The linear first-order polynomial approximation of the response function is

(5) $$\begin{array}{c} f=\beta_{0}+\overset{n}{\underset{i=1}{\sum }}\beta_{i}x_{i}+\varepsilon . \end{array}$$

The quadratic second-order polynomial approximation of the response function is

(6) $$\begin{array}{c} f=\beta_{0}+\overset{n}{\underset{i=1}{\sum }}\beta_{i}x_{i}+\overset{n}{\underset{i=1}{\sum }}\beta_{ii}x_{i}^{2}+\overset{n}{\underset{i=1}{\sum }}\overset{n}{\underset{j=1}{\sum }}\beta_{ij}x_{i}x_{j}+\cdots , \end{array}$$

where *β*_0_, *β*_*i*_, *β*_*ii*_, and *β*_*ij*_ are tuning parameters; *ε* is the error item; *n* is the number of model parameters.

The Appendix shows the nonlinear regression formulations of *Y*_1_ for NTSC, *Y*_2_ for NPSL, and *Y*_3_ for OP. The nonlinear regression formulations are significant for *Y*_1_ (*F* = 65.34, *P* < 0.0001), *Y*_2_ (*F* = 3099.554, *P* < 0.0001), and *Y*_3_ (*F* = 61.373, *P* < 0.0001). Also, the explained variances (*R* ^2^) are higher for *Y*_1_ (0.996), *Y*_2_ (0.999), and *Y*_3_ (0.993).

Furthermore, in order to obtain the optimal parameter settings, the GA was used to find the solution. A common method of terminating a GA is to test (after a specified number of generations) the quality and the convergence at the global optimum of the best members of the population against the tested problem definition. The roulette wheel approach was used as the selection procedure. The population size was set as 50, the mutation rate was set as 0.06, and the crossover rate was set as 0.5. The mutation rate and the crossover rate controlled the expected number of chromosomes to mate and the number of genes to mutate, respectively [23, 24]. The stopping condition of the GA procedure was set at 1,000 iterations or when the change in the previous 100 iterations was less than 1 percent. Parameters *A* and *B* were continuous variables, and Parameters *C* and *D* were discrete variables.

Table 2 shows the optimal parameter settings. For TSC, the safety stock was set as 100 (level 3), the maximum inventory level was set as 400 (level 1), the transportation capacity was set as 100 (level 1), and the reliability of the HSCS was set as 95 percent (level 3); the TSC was 1065479. For PSL, the safety stock was set as 100 (level 3), the maximum inventory level was set as 550 (level 2.498), the transportation capacity was set as 500 (level 3), and the reliability of the HSCS was set as 99 percent (level 1); PSL was 0.981. For the OP, the safety stock was set as 78 (level 1.810), the maximum inventory level was set as 400 (level 1), the transportation capacity was set as 100 (level 1), and the reliability of the HSCS was set as 99 percent (level 1); the OP was 0.962. To compare the results of decision behavior, when the decision-maker considers that the performance of the HSCS should depend on OP, the TSC would be 1076531.743 and the PSL would be 0.918. In this situation, the TSC (1076531.743) is 1.037 percent higher than the derived TSC (1065478.998) and the PSL (0.918) is 3.146 percent higher than the derived PSL (0.890) when the decision-maker considers that the HSCS performance should depend on TSC. Additionally, when the decision-maker considers that the performance of the HSCS should depend on the TSC, the TSC would be 1065478.998 and the PSL would be 0.890. In this situation, the TSC (1065478.998) is 13.144 percent lower than the derived TSC (1205528.075) and the PSL (0.890) is 10.225 percent lower than the derived PSL (0.981) when the decision-maker considers that the HSCS performance should depend on the PSL. Finally, when the decision-maker considers that the HSCS performance should depend on the PSL, the TSC would be 1205528.075 and the PSL would be 0.981. In this situation, the TSC (1205528.075) is 11.983 percent higher than the derived TSC (1076531.743) and the PSL (0.981) is 6.863 percent higher than the derived PSL (0.918) when the decision-maker considers that the performance of the HSCS should depend on OP. Hence, from the above comparison of decision behavior results, the HSCS's OP for the parameter settings is the superior choice because the TSC is 1076531.743, which is 1.037 percent higher than the derived TSC when the decision-maker considers that the HSCS performance should depend on the TSC. The PSL is 0.918, which is 6.754 percent lower than the derived PSL when the decision-maker considers that the HSCS performance should depend on the PSL.

## 3. Sensitivity Analysis

**Figure 1:**
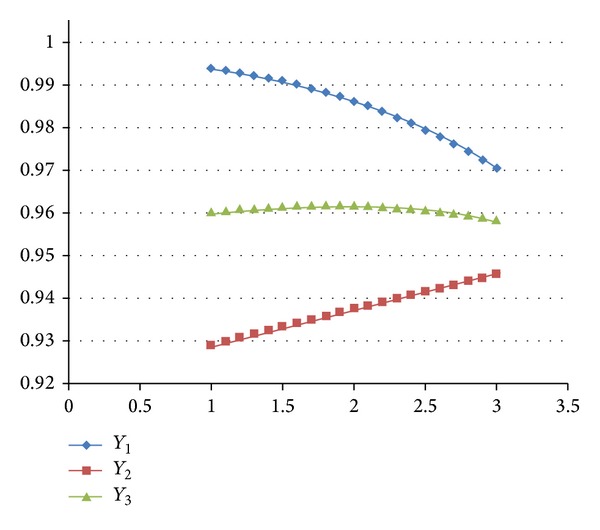
The changed safety stock levels affecting *Y*_1_, *Y*_2_, and *Y*_3_.

**Figure 2:**
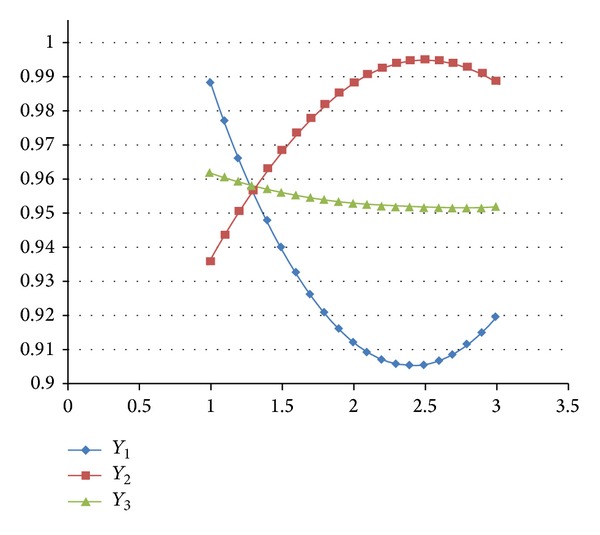
The changed maximum inventory levels affecting *Y*_1_, *Y*_2_, and *Y*_3_.

The sensitivity analysis was based on the most robust parameter settings when the performance of the HSCS depends on OP. The safety stock is from 70 (level 1) units to 100 (level 3) units, and the other parameter settings are fixed. The results (see Figure 1) show that the *Y*_1_ curve decreases from 0.993 to 0.970, which means that the TSC increased. The *Y*_2_ curve increases from 0.929 to 0.945, which means that the PSL increased. The *Y*_3_ curve slightly increases from 0.960 to 0.962 (the safety stock level is 1.810, i.e., 78 units) and then slightly decreases to 0.958. In addition, the maximum inventory level ranges from 400 units (level 1) to 600 units (level 3), and the other parameter settings are fixed.  Figure 2 shows that the *Y*_1_ curve decreases from 0.988 to 0.905 (maximum inventory level is 2.4, i.e., 540 units), which means that the TSC increased. The *Y*_1_ curve subsequently increases to 0.920, which means that the TSC decreased. The *Y*_2_ curve increases from 0.936 to 0.994 (maximum inventory level is 2.4, i.e., 540 units), which means that the PSL increased. It then decreases to 0.989, which means that the PSL likewise decreased. The *Y*_3_ curve decreases slightly from 0.962 to 0.952.

When the transportation capacity increased from level 1 and level 2 to level 3, the results (see Table 3) show that *Y*_1_ decreases, which means that the TSC increased. *Y*_2_ decreases slightly, which means that the PSL decreased slightly. *Y*_3_ decreases.  Table 4 shows that when the reliability of the HSCS increased from level 1 and level 2 to level 3, the *Y*_1_ in level 1 is 0.988, in level 2 is 0.984, and in level 3 is 0.987. *Y*_2_ decreases, which means that the PSL similarly decreased. *Y*_3_ decreases slightly.

**Figure 3:**
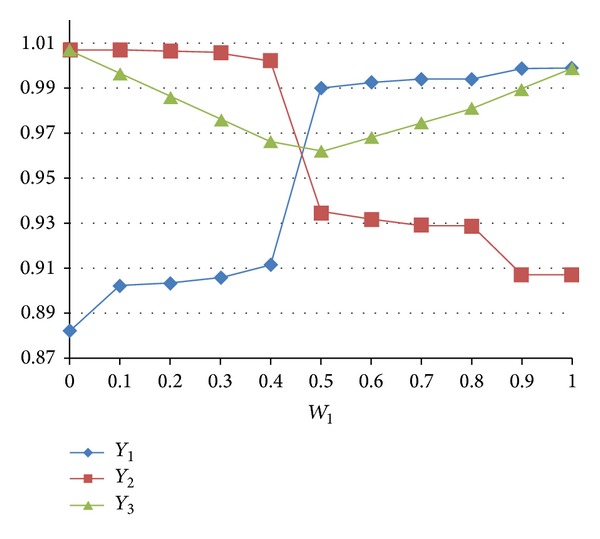
The changed weights affecting *Y*_1_, *Y*_2_, and *Y*_3_.

In addition, to compare the trade-off effect, when different weight combinations are formed, OP = *W*_1_∗NTSC + *W*_2_∗NPSL where *W*_1_ + *W*_2_ = 1; the results are shown in Figure 3 and as follows.

1. When *W*_1_ is from 0.1 to 0.4, *Y*_1_ increases from 0.902 to 0.912 and *Y*_2_ decreases from 1.007 to 1.002. The parameters' setting for the safety stock is level 3, the maximum inventory level is from 2.453 to 2.084, the transportation capacity is level 1, and the reliability of HSCS is level 1.
2. When *W*_1_ is from 0.4 to 0.5, *Y*_1_ immediately increases from 0.912 to 0.989 and *Y*_2_ immediately decreases from 1.00 to 0.934. The parameters' setting for the safety stock level is 1.615, the maximum inventory level is level 1, the transportation capacity is level 1, and the reliability of HSCS is level 1.
3. When *W*_1_ is from 0.5 to 0.8, *Y*_1_ increases from 0.989 to 0.994 and *Y*_2_ decreases from 0.934 to 0.929. The parameters' setting for the safety stock level is from 1.615 to 1.00, the maximum inventory level is level 1, the transportation capacity is level 1, and the reliability of HSCS is level 1.
4. When *W*_1_ is from 0.9 to 1.0, the *Y*_1_ is 0.998, and *Y*_2_ is 0.907. The parameters' setting for the safety stock is level 3, the maximum inventory level is level 1, the transportation capacity is level 1, and the reliability of HSCS is level 1.
5. When OP is set to $\sqrt{\text{NTSC}*\text{NPSL}}$ as the ideal function, *Y*_3_ = 0.962, and the safety stock is level 1.810, the maximum inventory level is level 1, the transportation capacity is level 1, and the reliability of HSCS is level 1. This situation is near the situation when OP is set to *W*_1_∗NTSC + *W*_2_∗NPSL; in the (*W*_1_, *W*_2_). *W*_1_ is set between 0.4 to 0.5, and *W*_2_ is between 0.5 to 0.6. It means that the ideal function weights *W*_1_ and *W*_2_ are set between 0.4 to 0.5 and between 0.5 to 0.6. Hence, when W_1_ is less than the ideal function weight, the performance *Y*_2_ is better than *Y*_1_. When *W*_1_ is larger than the ideal function weight, the performance *Y*_1_ is better than *Y*_2_.

## 4. Conclusion

In this paper, the authors proposed a method of setting parameters to design a robust HSCS. The full-factor experimental design was used to obtain simulation data for the HSCS. The RSM was applied here to obtain a regression model that shows how the parameter settings affect the HSCS's performance. Also, GA was used to search for the solution to the problem of identifying optimal parameter settings. The results show that the best choice for the HSCS parameter settings is when the decision-maker simultaneously takes the TSC and the PSL into consideration; in this scenario, the TSC is only 1.037 percent higher than the derived TSC when the decision-maker considers that the HSCS performance should depend on the TSC. The PSL is only 6.754 percent lower than the derived PSL when the decision-maker considers that the HSCS performance should depend on the PSL. Also, the adjustable strategies coming from the results of sensitivity analysis show that when the OP is considered and the HSCS parameters' setting is set in the optimization (the safety stock: 78 units (level 1.810); the maximum inventory level: 400 units (level 1); the transportation capacity: 100 (level 1); the reliability of the HSCS: 99 percent (level 1)), if the safety stock is adjusted increasing/decreasing 1 unit, the TSC will be changed (increasing/decreasing) 0.077%, and PSL will be changed (increasing/decreasing) 0.053%. If the maximum inventory level is adjusted increasing from 400 units to 540 units, the TSC will increase 0.593% per unit and PSL will increase 0.414% per unit. In addition, if the maximum inventory level is adjusted increasing from 540 units to 600 units, the TSC will decrease 0.250% per unit and PSL will decrease 0.083% per unit. If the transportation capacity is adjusted to level 2 or level 3, the TSC will increase 2.1% or 13.8%, and PSL will decrease 0.000% or 0.002%. If the reliability of the HSCS is adjusted to level 2 or level 3, the TSC will increase 0.04% or 0.01%, and PSL will decrease 0.010% or 0.015%. Hence, the decision-makers can evaluate the changed TSC and PSL to adjust the parameters' level as adjustable strategies when dealing with practical problems. The concept of adjustable strategies will play an important role in future research on HSCS.

In addition, to compare the trade-off effect between TSC and PSL, the results show that when the *W*_1_ is less than the ideal function weight, the PSL will be stronger to affect the OP than the TSC. When the *W*_1_ is larger than the ideal function weight, the TSC will be stronger to affect OP than the PSL. Future study may consider using any MOO (multiobjective optimization) method (epsilon constraint, normal boundary intersection) to obtain the optimal parameter settings for the HSCS and to evaluate the performance of the HSCS.

## Figures and Tables

**Table 1:** The parameter levels in this study.

Parameters	Value		
	Level 1	Level 2	Level 3
*A*: safety stock	70	80	100
*B*: maximum inventory level	400	500	600
*C*: transportation capacity	100	230	500
*D*: the reliability of HSCS	99%	97%	95%

**Table 2:** The optimal parameter settings for  *Y*_1_, *Y*_2_, and *Y*_3_.

	*A*	*B*	*C*	*D*	Response
Total system cost (*Y*_1_)	3	1	1	3	0.998
Patient safety level (*Y*_2_)	3	2.498	3	1	1.000
Overperformance (*Y*_3_)	1.810	1	1	1	0.962

**Table 3:** The transportation capacity levels affecting *Y*_1_, *Y*_2_, and *Y*_3_.

Transportation capacity		*Y*_1_	*Y*_2_	*Y*_3_
Level	Units			
3	100	0.850	0.934	0.890
2	230	0.967	0.936	0.951
1	500	0.988	0.936	0.962

**Table 4:** The reliability of the HSCS levels affecting *Y*_1_, *Y*_2_, and *Y*_3_.

The reliability of the HSCS		*Y*_1_	*Y*_2_	*Y*_3_
Level	Reliability			
3	95%	0.987	0.921	0.953
2	97%	0.984	0.926	0.955
1	99%	0.988	0.936	0.962

## Appendix

### Regression Formulations

Consider

(A.1) Y1=1.524+0.146A−1.020B−0.264C−0.968D −0.084AB−0.109AC+0.137AD+0.867BC +1.372BD+0.852CD−0.069A2+0.299B2 +0.020C2+0.262D2−0.197ABD−0.058ACD −1.309BCD+0.073A2B+0.026A2C+0.011A2D +0.017AB2+0.022AC2−0.036AD2−0.288B2C −0.392B2D−0.180BC2−0.287BD2−0.181C2D −0.231CD2+0.107ABCD−0.018A2B2−0.023A2BC −0.010A2BD+0.001A2C2−0.006A2CD−0.003A2D2 +0.005AB2C+0.060AB2D+0.050ABD2+0.015ACD2 +0.065B2C2+0.390B2CD+0.100B2D2+0.282BC2D +0.207BCD2+0.050C2D2+0.005A2B2C −0.001A2BC2+0.008A2BCD+0.003A2BD2 −0.002A2C2D+0.004A2CD2−0.038AB2CD −0.016AB2D2−0.028ABCD2−0.085B2C2D −0.100B2CD2+0.003A2BCD2+0.010AB2CD2 +0.020B2C2D2−0.012BC3D2,

(A.2) Y2=0.648+0.280A+0.360B+0.018C+0.194D −0.348AB−0.329AD−0.011BC−0.262BD −0.011CD−0.091A2−0.081B2−0.006C2 −0.046D2+0.419ABD+0.004BCD+0.118A2B +0.114A2D+0.084AB2+0.075AD2+0.002B2C +0.063B2D+0.004BC2+0.057BD2+0.004C2D +0.001CD2−0.029A2B2−0.145A2BD−0.027A2D2 −0.102AB2D−0.090ABD2−0.013B2D2−0.001BC2D +0.035A2B2D+0.032A2BD2+0.021AB2D2 −0.007A2B2D2,

(A.3) Y3=1.091+0.053A−0.362B−0.098C−0.279D +0.001AB−0.050AC−0.016AD+0.406BC +0.424BD+0.317CD−0.010A2+0.117B2 +0.020C2+0.080D2−0.007ABC+0.009ABD +0.018ACD−0.520BCD+0.004A2B+0.003A2C +0.001A2D−0.003AB2+0.009AC2+0.001AD2 −0.140B2C−0.127B2D−0.106BC2−0.113BD2 −0.089C2D−0.093CD2−0.014ABCD−0.001A2B2 −0.001A2BD+0.002A2CD−0.001A2D2 +0.005AB2C−0.002AB2D−0.002AC2D +0.038B2C2+0.159B2CD+0.032B2D2+0.144BC2D +0.136BCD2+0.024C2D2+0.001A2C2D +0.002AB2CD+0.002ABC2D−0.044B2C2D −0.040B2CD2−0.036BC2D2+0.010B2C2D2.
